# Multicompartment body composition analysis in older adults: a cross-sectional study

**DOI:** 10.1186/s12877-023-03752-1

**Published:** 2023-02-09

**Authors:** Ana Claudia Rossini-Venturini, Lucas Veras, Pedro Pugliesi Abdalla, André Pereira dos Santos, Márcio Fernando Tasinafo-Junior, Leonardo Santos Lopes da Silva, Thiago Cândido Alves, Eduardo Ferriolli, Vicente Romo-Perez, Jose Luis Garcia-Soidan, Jorge Mota, Dalmo Roberto Lopes Machado

**Affiliations:** 1 College of Nursing at Ribeirão Preto, University of São Paulo, Avenue of Bandeirantes nº 3900, University Campus - Monte Alegre, Ribeirão Preto-SP, Brazil; 2 Study and Research Group in Anthropometry, Training, and Sport (GEPEATE), São Paulo, Brazil; 3 The Research Centre in Physical Activity, Health, and Leisure (CIAFEL), University of Porto, Porto, Portugal; 4 Laboratory for Integrative and Translational Research in Population Health (ITR), University of Porto, Porto, Portugal; 5 School of Physical Education and Sport of Ribeirão Preto, University of São Paulo, Ribeirão Preto, Brazil; 6 Ribeirão Preto Medical School, University of São Paulo, Ribeirão Preto, Brazil; 7 Laboratório de Investigação Médica em Envelhecimento (LIM-66), Serviço de Geriatria, Hospital das Clínicas HCFMUSP, Faculdade de Medicina, Universidade de São Paulo (USP), São Paulo, Brazil; 8 Faculty of Education and Sport Sciences, University of Vigo, Vigo, Spain; 9 ESEC - Universidade do Algarve. , Campus da Penha, Faro, Portugal

**Keywords:** Aging, DXA, Equation, Fat mass, Bone mineral content, ALST

## Abstract

**Background:**

During aging, changes occur in the proportions of muscle, fat, and bone. Body composition (BC) alterations have a great impact on health, quality of life, and functional capacity. Several equations to predict BC using anthropometric measurements have been developed from a bi-compartmental (2-C) approach that determines only fat mass (FM) and fat-free mass (FFM). However, these models have several limitations, when considering constant density, progressive bone demineralization, and changes in the hydration of the FFM, as typical changes during senescence. Thus, the main purpose of this study was to propose and validate a new multi-compartmental anthropometric model to predict fat, bone, and musculature components in older adults of both sexes.

**Methods:**

This cross-sectional study included 100 older adults of both sexes. To determine the dependent variables (fat mass [FM], bone mineral content [BMC], and appendicular lean soft tissue [ALST]) whole total and regional dual-energy X-ray absorptiometry (DXA) body scans were performed. Twenty-nine anthropometric measures and sex were appointed as independent variables. Models were developed through multivariate linear regression. Finally, the predicted residual error sum of squares (PRESS) statistic was used to measure the effectiveness of the predicted value for each dependent variable.

**Results:**

An equation was developed to simultaneously predict FM, BMC, and ALST from only four variables: weight, half-arm span (HAS), triceps skinfold (TriSK), and sex. This model showed high coefficients of determination and low estimation errors (FM: R^2^_adj_: 0.83 and SEE: 3.16; BMC: R^2^_adj_: 0.61 and SEE: 0.30; ALST: R^2^_adj_: 0.85 and SEE: 1.65).

**Conclusion:**

The equations provide a reliable, practical, and low-cost instrument to monitor changes in body components during the aging process. The internal cross-validation method PRESS presented sufficient reliability in the model as an inexpensive alternative for clinical field use.

## Background

Muscle, fat, and bone are three main components of interest in the body composition (BC) field [1]. The aging process involves proportional changes in these components [1] due to decreased levels of anabolic steroids and sex hormones [2]. These alterations in the older adults’ BC have a great impact on their health and quality of life [3]. Skeletal muscle mass (SMM) has various essential physiological functions in humans and its maintenance is important to keep the body healthy, especially during aging. Thus, the reduction of SMM impairs muscle strength, and functional capacity, increasing the chances of morbidity and mortality [4]. As a large proportion of SMM (≅ 74%) is found in the extremities, the appendicular lean soft tissue (ALST) is a representative measure of the SMM [5]. In addition, ALST is used to identify sarcopenia [6]. In turn, the bone mineral content (BMC) presents important variations throughout the older’ life. Peak BMC occurs in the third decade of life and declines over the years [7]. This reduction is similar in men and women before 50 years of age, but after this, the differences become very distinct among women because of menopause [8]. This skeletal reduction restrains bone strength and can cause osteopenia and osteoporosis. Osteoporosis increases the risk of fractures and is considered the main consequence of the disease [9]. Meanwhile, fat mass (FM) presents an increases during aging [10]. From 70 years old, the FM increases (7.5%) in a similar way for both sexes [11], becoming one of the main risk factors for chronic diseases [12], colon cancer [13], physical function [14] and mortality [15]. In this sense, changes in ALST, BMC, and FM during senescence have a great impact on their health [16], quality of life, and physical functional [17]. To monitor this BC variability, simple and low-cost methods are required [18].

Several equations to predict BC using anthropometric measurements have been developed to determine FM and fat-free mass (FFM). However, these models have limitations regarding the estimation of adult older’s body density (BD) and BC [19]. The traditional bi-compartmental (2-C) model assumes that there is a linear relationship between subcutaneous fat, total fat, and BD. However, the correlation between total and subcutaneous body fat decreases with age [20]. Perhaps it is due to; 1) the redistribution of FM from the extremities to the visceral area, and 2) due to fat infiltration in the SMM. Thus, there is an overestimation of the BD, and consequently, the FM is underestimated [21]. Another worrying limitation is to assume a constant density of 0.9007 g/cm^3^ and 1.100 g/cm^3^ for the FM [22] and FFM [23], respectively. However, the natural aging process causes progressive bone demineralization [24] and changes in the hydration of the FFM, causing a decrease in its density [25] which also affects the FM estimate [24]. Furthermore, these 2-C equations do not evaluate other components, such as ALST and BMC, fundamental components in older adults.

From methodological advances it is necessary to analyze BC in a more precise and detailed way [26]. Among imaging analysis methods, dual-energy X-ray absorptiometry (DXA) is widely used because its offers advantages such as low cost, speed of measurement, noninvasive, efficiency in the simultaneous determination of several components in a single scan [27], and their radiation exposure are considered small and safe for repeated measures (< 1 mrem for whole-body scans) [28]. Furthermore, DXA is considered a 3-C model [29], once it can accurately measure FM, BMC, and ALST [30]. However, BC assessment with sophisticated equipment such as DXA is restricted to specific professionals, requiring a specialized structure. Then, due to anthropometric measurements are simple and with a low cost associated [31], their use has been presented as valid alternatives for estimating BC in a multicompartmental approach in children and adolescents of both sexes [32, 33]. So, the objective of this study was to propose and validate a multi-compartmental anthropometric model for the prediction of fat, bone, and musculature components in older adults of both sexes. Our hypothesis is that BC can be estimated through anthropometric measurements.

## Methods

### Design and study population

In this study, we adopted a cross-sectional design to develop and validate a multicomponent anthropometric model to simultaneously estimate LST, BMC, and FM. The study was conducted from October 2016 to May 2017. The study sample was derived from physically independent community-dwelling older adults in a city in southeastern Brazil. The inclusion criteria were: adults aged 60–85 years, of both sexes, who walk independently. The exclusion criteria were: the presence of diseases that restrict mobility or muscle strength; absence of unstable cardiovascular condition; acute infection; tumor; back pain; prostheses, individuals with a diagnosis of cancer or uncontrolled diseases, who presented sequel of stroke, experienced a weight loss more than three kilograms (kg) in the last 3 months, had a cognitive limitation that restricts understanding and taking tests, who did not complete all the stages or desired to withdraw from the study.

The study was approved by the Ethical Review Board of Hospital das Clinicas at the Medical School of the University of São Paulo (HC-FMRP/USP), following the ethical guidelines outlined in the 1975 Helsinki Declaration. Written informed consent was obtained from all individuals included in the study, after a brief explanation of the study objectives and evaluations. This manuscript followed the guidelines from The Strengthening the Reporting of Observational Studies in Epidemiology (STROBE) conference list.

The sample size calculation was considered the desired maximum error (ε) and degree of confidence (Zy), previously knowing the population variability (σ^2^) [34]. For this, we used the variable with the greatest variability (FM; SD = 8.7 kg) expected for such a population [35]. Once the predetermined error estimate (ε ≤ 1.8 kg) and maximum desired error (5%) the ideal n for the study [34] was defined (*n* = 90).

### Study protocol

A multidisciplinary health-trained team (nurses, nutritionists, pharmacists, physical education professors, physicians, and physiotherapists) performed data collection. All procedures, for each participant, were completed during one visit to the laboratories at the HC-FMRP/USP. Participants came to the laboratory after an overnight fast (8 h fast), abstaining from vigorous exercises, and no caffeine and alcohol during the preceding 24 h. Before the measurements, the subjects were asked to empty their bladders. A total-body DXA scan was executed according to the manufacturer's guidelines. The anthropometric measures were taken according to the literature guidelines [36], whose procedures are summarized below.

### The dependent variables

Whole and regional BC were determined by DXA (Hologic® scanner, model QDR4500W; version 11.2, Bedford, MA). The DXA measurements included absolute values of appendicular lean soft tissue (ALST, kg), bone mineral content (BMC, kg), and fat mass (FM, kg), considered dependent variables. As the BMC represents the gray portion of bone, the bone adjustment was performed by multiplying the BMC by 1.0436 [37]. The ALST was obtained through the sum of the lean soft tissue (LST) of the lower and upper limbs on both sides [38]. The DXA measurements were electronically transferred to an external HD and organized into a general data sheet without manual typing.

### The independent variables

The participant’s body mass and height were measured with a digital scale (Filizola® (model Personal, *Campo Grande*, MS) and a hall fixed stadiometer (Sanny® Professional – ES2020), respectively. The skinfolds (*n* = 09; subscapular, triceps, biceps, media axillary, pectoral, suprailiac, vertical abdominal, media thigh and calf) were measured with Lange caliper with precision in mm, on the right side of the body in the regions. The circumferences (*n* = 08; chest, arm, forearm, waist, abdominal, hip, medial thigh and calf) were measured using inelastic and inextensible tape (Sanny®). The girths (*n* = 08; bi-acromial, bi-iliac, bi-trochanteric, bi-malleolar, biepicondylar humerus, bi-styloid, biepicondylar femur and transverse thoracic) were measured with Pachymeter (Sanny®). In addition, knee height and half-arm span (HAS) were measured using a Sanny® segmometer. All anthropometric measurements were performed by the same trained evaluator. All these procedures followed conventional standardization [39]. The anthropometric measurements of our laboratory remain within the limits of reliability [33].

### Statistical analysis

The basic analysis involved descriptive statistics using measures of central tendency to describe the characteristics of the sample. To verify the data normality, the Shapiro–Wilk test was applied. Comparisons between sex were performed using Student’s t-test for independent samples. For the Multicompartmental anthropometric equation development, we adopted previous procedures [32, 33], briefly described below.

Through the determination of 30 independent variables plus the sex for the prediction of the 3 dependent variables, the multivariate regression model (nYm = nX(r + 1) (*r* + 1) βm + nεm) by diagonal mutual analysis, parameter estimation, and the least squares errors method was used [40] by R Statistical Software (version 4.1.2, R Foundation for Statistical Computing, Vienna, Austria). The criteria for selection and reduction of independent variables followed the following steps: a) factor analysis and model adequacy (Kaiser–Meyer–Olkin) and Sphericity test (Bartlett) were performed to verify the suitability of the sample; b) univariate linear regression to determine all common independent variables for each dependent variable (ALST, BMC, and FM), with significantly less than 5%; c) multivariate linear regression to estimate the parameters and Pillai approximation method for showing possible variables exclusions; d) testing of the remaining model (enter—univariate method), with estimated values of VIF (< 10.0) and multicollinearity (L < 1000) maximum permitted; e) adjustments by Pillai approach to testing the F values; f) as the variable sex is a categorical variable, it could not enter in the factor analysis. However, it will be added to the multivariate model due to its theoretical relevance and assumption of improving the model; g) then multivariate β parameters were determined, with the proposition of equations and residual distribution for each dependent variable; h) Akaike information criterion (AIC) statistic to ensure greater quality and simplicity of the statistical model. The details of the statistical procedures have been previously described in adolescents of both sexes [32, 33].

Finally, the predicted residual error sum of squares (PRESS) statistic was used to measure the effectiveness of the predicted equations for each dependent variable. The procedure may be understood as design efficiency in estimating the actual parameters by a virtual simulation that is, from the exclusion of an observation, equations are proposed with the remaining sample and replicated through cross-validation for each participant that was excluded. For validation, we follow the following steps: a) the correlation coefficients were estimated between predicted and measured values and b) cross-validation by PRESS method, coefficients of determination (Q^2^_PRESS_), and error (S_PRESS_) for each dependent variable (ALST, BMC, and FM) [40].

## Results

Table 1 shows the anthropometric and BC measures of the eligible participants. The means of all variables are within the confidence interval (95% CI), within the range limits for normal trends of distribution. Men were statistically taller, heavier, larger, and longer in most comparisons with women. Also had higher values of ALST, BMC, and residual mass. On other hand, women presented higher skinfolds, fat mass, and circumferences of hip and thigh values (*p* < 0.05).

**Table 1:** Descriptive values of anthropometric and body composition variables in older adults, difference test by sex

	Men (*n* = 31)					Women (*n* = 69)					
	Mean (SD)	95% CI		Variance	Min—Max	Mean (SD)	95% CI		Variance	Min—Max	*p*
		IL	UL				IL	UL			
Age (years)	72 (7.6)	69.2	74.8	57.8	60.0 – 88.0	69.8 (5.9)	68.4	71.3	35.4	60.0 – 85.0	0.133
*Anthropometric variables*											
Height (cm)	168.0 (8.0)	165.0	170.9	64.0	150.5 – 188.0	156.2 (6.0)	154.8	157.6	35.4	145.0 – 174.0	< 0.001
Weight (kg)	71.7 (13.3)	66.9	76.6	177.8	42.0 – 108.0	65.8 (11.4)	63.0	68.5	130.9	39.0—102.0	0.022
Knee height (cm)	53.6 (2.6)	52.6	54.5	7.0	49.1—61.8	49.6 (2.1)	49.1	50.1	4.5	45.0—54.4	< 0.001
Half-arm span (cm)	87.4 (4.7)	85.6	89.1	21.8	78.7 – 101.0	80.9 (3.7)	79.9	81.8	13.9	69.1—89.5	< 0.001
*Skinfold (mm)*											
Subscapular skinfold	23.2 (8.5)	20.0	26.3	72.3	6.0 – 37.0	28.3 (8.8)	26.2	30.4	77.0	8.0 – 47.0	0.007
Triceps skinfold	15.1 (5.8)	13.0	17.3	34.0	4.0 – 26.0	25.8 (6.9)	24.2	27.5	48.0	9.0 – 46.0	< 0.001
Biceps skinfold	8.1 (3.5)	6.8	9.4	12.2	3.0 – 15.0	15.2 (5.3)	13.9	16.5	28.6	5.0 – 34.0	< 0.001
Media axillary skinfold	18.5 (7.6)	15.8	21.3	57.4	4.0 – 30.0	23.7 (6.8)	22.1	25.4	46.5	5.0 – 41.0	0.001
Pectoral skinfold	16.9 (5.4)	15.0	18.9	28.9	4.0 – 26.0	14.7 (6.4)	13.2	16.2	40.5	4.0 – 40.0	0.094
Suprailiac skinfold	19.7 (9.8)	16.1	23.3	95.5	5.0 – 39.0	29.3 (7.9)	27.4	31.2	63.0	8.0 – 50.0	< 0.001
Vertical abdominal skinfold	25.9 (8.3)	22.8	28.9	68.5	5.0 – 39.0	33.6 (8.6)	31.6	35.7	74.5	6.0 – 55.0	< 0.001
Media Thigh skinfold	18.1 (7.5)	15.4	20.9	56.0	5.0 – 40.0	32.2 (11)	29.6	34.8	120.1	9.0 – 65.0	< 0.001
Calf skinfold	11.9 (6.4)	9.5	14.3	41.4	2.0 – 34.0	23.8 (7.4)	22.0	25.6	55.2	6.0 – 45.0	< 0.001
*Circumference (cm)*											
Chest circumference	97.8 (9.2)	94.4	101.1	84.5	82.0—116.5	92.9 (7.6)	91.1	94.8	58.2	72.0—115.5	0.007
Arm circumference	28.8 (3.5)	27.6	30.1	12.0	20.0 – 36.0	29.9 (3.8)	29.0	30.8	14.1	22.0—40.8	0.178
Forearm circumference	26.0 (2.0)	25.2	26.7	3.8	20.0 – 30.0	23.8 (2.3)	23.3	24.4	5.1	19.0 – 30.0	< 0.001
Waist circumference	92.2 (11.1)	88.1	96.2	122.8	68.5 – 115.0	86.2 (10.0)	83.8	88.6	100.6	65.0 – 113.0	0.009
Abdominal circumference	96.1 (10.9)	92.1	100.1	118.5	68.0—115.5	95.4 (10.9)	92.8	98.0	119.7	70.0 – 120.0	0.766
Hip circumference	96.8 (6.6)	94.4	99.2	43.1	80.0—111.5	100.6 (9.0)	98.5	102.8	81.7	81.5 – 128.0	0.038
Medial thigh circumference	47.1 (5.6)	45.1	49.2	31.7	32.0 – 57.0	52.8 (6.3)	51.3	54.3	40.3	37.5 – 69.0	< 0.001
Calf circumference	35.6 (3.5)	34.3	36.9	12.1	26.5—42.5	34.9 (3.0)	34.2	35.6	8.7	25.2 – 42.0	0.268
*Girth (cm)*											
Bi-acromial breadth	39.9 (2.7)	38.9	40.9	7.1	33.6—44.1	37 (2.0)	36.6	37.5	4.1	32.9 – 45.0	< 0.001
Bi-iliac breadth	31.3 (2.6)	30.3	32.3	6.9	28.1—39.8	30.8 (2.1)	30.2	31.3	4.3	26.2 – 37.0	0.268
Bi-trochanteric breadth	33.8 (1.7)	33.2	34.5	2.9	31.4—39.7	33.3 (2.3)	32.8	33.9	5.2	28.7—39.9	0.289
Bi-malleolar breadth	7.0 (0.5)	6.8	7.2	0.3	6.0—8.2	6.3 (0.4)	6.2	6.4	0.1	5.4—7.4	< 0.001
Biepicondylar humerus breadth	6.7 (0.5)	6.5	6.9	0.2	5.7—7.7	5.8 (0.5)	5.7	5.9	0.2	4.8—6.8	< 0.001
Bi-styloid breadth	5.7 (0.4)	5.6	5.9	0.2	5.2—6.8	5.1 (0.4)	4.9	5.2	0.1	4.4—6.4	< 0.001
Biepicondylar femur breadth	9.7 (0.7)	9.5	9.9	0.4	7.9—11.6	9.4 (0.8)	9.2	9.6	0.6	8.0—12.4	0.062
Transverse thoracic breadth	30.8 (2.6)	29.9	31.8	6.6	24.9—36.7	27.7 (1.8)	27.3	28.2	3.3	22.3—31.4	< 0.001
*Body composition* *(kg)*											
ALST	20.7 (4.1)	19.2	22.2	16.5	12.6 – 32.5	14.6 (2.6)	14.0	15.2	6.5	9.2 – 22.5	< 0.001
Fat mass	21.7 (6.9)	19.2	24.2	47.4	7.7—33.6	27.9 (7.2)	26.1	29.6	51.7	13.6—47.9	< 0.001
Bone mineral content	2.6 (0.6)	2.3	2.8	0.3	1.4 – 3.8	2.0 (0.3)	1.9	2.1	0.1	1.4—3.1	< 0.001
Residual mass	22.8 (3.9)	21.4	24.2	15.1	15.8 – 36.2	18.5 (2.8)	17.8	19.2	8.1	12.7 – 27.7	< 0.001

*CI* Confidence interval, *SD* Standard deviation, *UL* Upper limit, *IL* Inferior limit, *Min–Max* Minimum–maximum, *ALST* Appendicular lean soft tissue

The Kaiser–Meyer–Olkin test showed the sample adequacy and resulted in a value of 0.885, classified as meritorious [41] and the Barlett sphericity test yielded a Χ^2^ of 3368.04 (*p* < 0.001), indicating homogeneous variance between groups. From the univariate regression (stepwise), the number of remaining variables to ALST (*n* = 08), FM (*n* = 05), and BMC (*n* = 06) showed high r^2^_adj_ (0.68 to 0.88) for the independent common variables for the three dependents variables (Table 2). In bold, variables with statistically significant coefficients (*p* < 0.05), common in at least two of the dependent variables are shown.

**Table 2:** Univariate regression for selecting common independent variables at least twice (bold)

**Appendicular lean soft tissue**			**Fat mass**			**Bone mineral content**		
**Variables**	**Coefficient**	***p***	**Variables**	**Coefficient**	***p***	**Variables**	**Coefficient**	***p***
**Pectoral skinfold (mm)**	0.13018	< 0.001	Media axillary skinfold (mm)	0.16083	0.012	Bi-styloid breadth (cm)	0.210010	0.020
**Weight (kg)**	0.13214	< 0.001	**Pectoral skinfold (mm)**	-0.24240	< 0.001	Waist circumference (cm)	-0.030637	0.001
Knee height (cm)	0.23779	0.009	**Media thigh skinfold (mm)**	0.12626	0.008	**Triceps skinfold (mm)**	-0.020330	0.001
Bi-acromial breadth (cm)	0.30532	0.001	**Weight (kg)**	0.35620	< 0.001	**Weight (kg)**	0.038668	< 0.001
**Biepicondylar humerus breadth (cm)**	1.22979	< 0.001	**Half-arm span (cm)**	-0.29770	0.002	**Half-arm span (cm)**	0.032912	0.005
Calf circumference (cm)	0.26318	0.001				Medial thigh circumference (cm)	-0.028782	0.001
**Media thigh skinfold (mm)**	-0.07881	< 0.001						
**Triceps skinfold (mm)**	-0.09662	0.003						
R^2^ = 0.88			R^2^ = 0.85			R^2^ = 0.68		

R^2^: coefficient of determination variables with statistically significant coefficients (*p* < 0.05), common in at least two of the dependent variables are shown in bold

Next, a multivariate linear regression model was developed, simultaneously for the three dependent variables from variables selected in the univariate models. The categorical sex variable has not been previously tested in the models; however, it was added to the multivariate procedure due to its theoretical relevance, as demonstrated by their significant differences in Table 1. The coefficients, variance inflation factor (VIF), Pillai’s trace, and precision and cross-validation results are shown in Table 3. The equations presented below in Table 3, should be also presented as:

$${\mathrm{ALST}}_{[\mathrm{kg}]} = \left({\mathrm{0,19336 x weight}}_{[\mathrm{kg}]}\right) + \left({\mathrm{0,20139 x half}-\mathrm{arm span}}_{[\mathrm{cm}]}\right)+ \left({-\mathrm{ 0,04796 x triceps skinfold}}_{[\mathrm{mm}]}\right) + \left({-\mathrm{ 3,16675 x sex}}_{[\mathrm{women}=1;\mathrm{ men}=0]}\right) -\mathrm{10,21376}.$$

$${\mathrm{FM}}_{[\mathrm{kg}]} = \left({\mathrm{0,50239 x weight}}_{[\mathrm{kg}]}\right) + \left({-\mathrm{ 0,40498 x half}-\mathrm{arm span}}_{[\mathrm{cm}]}\right) +\left({\mathrm{0,17292 x triceps skinfold}}_{[\mathrm{mm}]}\right) + \left({\mathrm{4,73524 x sex}}_{[\mathrm{women}=1;\mathrm{ men}=0]}\right) +\mathrm{17,85412}.$$

$${\mathrm{BMC}}_{[\mathrm{kg}]} = \left({\mathrm{0,01912 x weight}}_{[\mathrm{kg}]}\right) + \left({\mathrm{0,02944 x half}-\mathrm{arm span}}_{[\mathrm{cm}]}\right) + \left({-\mathrm{ 0,01267 x triceps skinfold}}_{[\mathrm{mm}]}\right) + \left({\mathrm{0,13021 x sex}}_{[\mathrm{women}=1;\mathrm{ men}=0]}\right) -\mathrm{1,21230}.$$

**Table 3:** Coefficients, precision, and validation of a multicomponent anthropometric model to estimate body composition in older adults

	Appendicular lean soft tissue	Fat mass	Bone mineral content	VIF	F	*p* (Pillai's trace)
***Coefficients***						
Intercept	-10.21376	17.85412	-1.21230			
Weight	0.19336	0.50239	0.01912	2.045	676.33	< 0.001
Half-arm span	0.20139	-0.40498	0.02944	2.049	9.53	< 0.001
Triceps skinfold	-0.04796	0.17292	-0.01267	2.544	4.17	0.008
Sex	-3.16675	4.73524	-0.13021	2.407	11.33	< 0.001
***Precision***						
R^2^	0.85	0.83	0.62			
R^2^_adjusted_	0.85	0.83	0.61			
SEE (kg)	1.65	3.16	0.30			
***Cross-validation***						
PRESS	287.00	1066.84	9.75			
Q^2^_PRESS_	0.84	0.81	0.58			
S_PRESS_ (kg)	0.18	0.34	0.03			

Measurement protocol

Weight was measured with the subject should stand still over the center of the platform with the body weight distributed between both feet, when the subject could use light indoor clothing, excluding shoes, long trousers, and sweaters

Half-arm span was measured with the subject standing and the feet together and with their back (for women) or chest (for men) against the wall. The arms were outstretched laterally and maximally at the level of the shoulders in contact with the wall, and with the hands flat and fingers outstretched. The tip of the middle (longest) finger (excluding the fingernail) of the right hand was kept in contact with the block, while the zero ends of the tape were set at the tip of the middle (longest) finger (excluding the fingernail) of the left hand

The triceps skinfold was measured in the midline of the posterior aspect of the arm, over the triceps muscle, at the point midway between the lateral projection of the acromion process of the scapula and the inferior margin of the olecranon process of the ulna. The level of measurement was determined by the distance between the lateral projection of the acromial process and the inferior border of the olecranon process of the ulna, determined with a tape measure. The subject was measured standing and the skinfold was measured with the arm hanging loosely. The triceps skinfold was picked up with the left thumb and index finger, and the tips of the calibers were applied to the skinfold at the marked level, approximately 1 cm proximal to the marked level

Sex: male = 0; female = 1

R^2^: coefficient of determination; R^2^_adjusted_: adjusted coefficient of determination; SEE: standard error to estimate; PRESS: sum of squares of residuals; Q^2^_PRESS_: press coefficient of determination; S_PRESS_: press standard error of estimate; VIF: variance inflation factor

Higher precision and cross-validation values of PRESS, Q^2^_PRESS,_ and low SEE_PRESS_ were found for each dependent variable (Table 3). These results showed that the models are valid to simultaneously predict ALST, FM, and BMC, with accordance close to “1” (Q^2^_PRESS_) and error close to “0” (S_PRESS_).

**Fig. 1:**
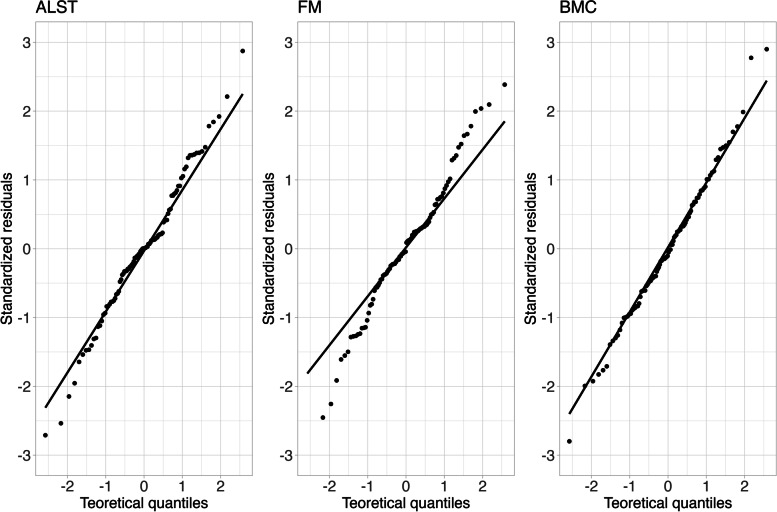
Model standardized residuals. ALST: appendicular lean soft tissue; FM: fat mass; BMC: bone mineral content

The model standardized residuals are normally distributed (*p* = 0.099) according to Fig. 1.

## Discussion

To the best of our knowledge, this is the first study that proposes a valid anthropometric model to simultaneously estimate FM, ALST, and BMC in older adults from a multicompartmental approach. DXA was used as a reference method due to its advantages in estimating all components by a single scan [42]. Our proposed model with three anthropometric variables plus sex showed high prediction coefficients and low errors to simultaneously predict ALST, FM, and BMC. Since BC is affected by sex [43], and changes in BC due to aging occur differently between men and women [44], the inclusion of the variable sex was made arbitrarily in the models generated in this study. Therefore, the current prediction equations are useful for estimating and monitoring ALST, FM, and BMC in older adults of both sexes.

Current anthropometric models to estimate BC in older adults have several limitations, causing errors in the estimation of BC. Furthermore, they have been developed using a bi-compartmental model (2-C) that determines FM and FFM [45–47], and this model is based on linear relationship between subcutaneous fat, total fat, and BD. However, this is not true, because during the aging process there is age-related adipose tissue redistribution that is, an accumulation of visceral and abdominal fat occurs [48]. Additionally, these equations do not evaluate ALST and BMC which are components that change during aging. The Lean equations [49] to estimate % body fat showed a coefficient of determination (r^2^) of 0.77 and 0.70 and a standard error of estimate (SEE) of 4.1% and 4.7% for older adults men and women, respectively. However, our results for FM determination showed a higher coefficient of determination (r^2^ = 0.83) and lower errors (SEE = 3,16 kg).

Progressive and metabolically unfavorable changes in BC have long been observed with aging [50]. In a prospective study that investigated age-dependent changes over two decades, the main results found were an increase in BM, BMI, and FM until the age of approximately 70 and 75 years, after these parameters start to decrease [51]. Regarding the changes in the SMM, the studies have shown a greater reduction in men than in women, with a more accentuated decline between 70 and 79 years old in both sexes [35, 50]. However, the pattern and rate of age-related changes in BC may vary by sex, ethnicity, physical activity level, and caloric intake [52].

DXA is the most popular technique for measuring BC [53] and it has been shown to be a reliable method of FFM during aging [54]. Furthermore, DXA may be considered the current reference technique for assessing SMM and BC in research and clinical practice [53]. A high correlation (*r* = 0.97) between DXA-measured ALST and SMM measured by magnetic resonance imaging (MRI) was reported for both men and women (18–92 years) [5]. In the same way, DXA-derived LST was found to be significantly correlated with MRI-measured SMM (*r* = 0.94; *p* ≤ 0.001) in older women [55]. In comparisons between DXA-measured FM and MRI-measured adipose tissue the associations were also high and significant (*r* = 0.99; *p* ≤ 0.001) for older women [55]. The principle of DXA depends on the property of X-rays to be attenuated in proportion to the composition and depth of the material the beam is crossed. The DXA scanner emits two different energy beams (40 and 70 keV). From the number of photons that are transmitted concerning the number detected the quantity of BMC and soft tissue (fat and FFM) can be determined [53]. Therefore, DXA can be used as a reference method to propose equations using anthropometry for clinical and professional practice [56]. The anthropometric measurements are performed in both the geriatric nutritional assessment and epidemiological studies because they are painless, safe, non-invasive, simple, and low-cost procedures, which permit the estimation of the body components and also the calculation of nutritional indicators using predictive equations [21]. The main anthropometric measurements used in older adults for this purpose are weight, height, calf and waist circumferences, as well as the triceps, biceps, subscapular and suprailiac skinfolds [21].

The current investigation has several strengths. As far as we know, this is the first study that proposes equations to estimate the main components of BC from the same anthropometric variables for older adults. This implies a reduction in the prediction error and facilitates its use in epidemiological studies. Another positive point is that we included the variable sex in the generated models, facilitating the application in large groups of both sexes. Despite all the research efforts in this study, there were still some limitations: for example, DXA is not a gold standard for older adults’ BC. However, the current state-of-the-art method for BC measurement in the four compartments model (4-C models) at the molecular level, as it includes the evaluation of the main FFM components, thus reducing the effect of biological variability. Nonetheless, it requires sophisticated and highly specialized technical equipment; it implies the propagation of measurement errors, difficult to apply in certain population groups, and is time-consuming. Furthermore, it has high costs, making it difficult to use on large samples [57]. Nevertheless, DXA represents a reference method for the assessment of human BC in the research field [42, 58] and it is widely considered the gold standard for BC assessment in clinical practice because of its advantages [56]. Another point to consider is that overnight fast impacts the hydration status and this can influence body composition measurement [59]. Moreover, reference values of BC assessed by DXA on adults over 60 years old are available from the National Health and Nutrition Examination Survey 1999–2004 and other studies on the local population [60]. Although it is a program designed to assess the health of adults and children in the United States, these reference values should be helpful in the evaluation of a variety of adult abnormalities involving fat, LST, and bone.

As hypothesized, using a multivariate regression model, simple anthropometric measures can be used to simultaneously estimate body components (ALST, FM, and BMC) in older adults of both sexes. As a practical simulation, an older adult male “A” with measurements of weight (66.3 kg), HAS (80.5 cm), TriSk (16 mm), and sex (0), when applied to our model, would have the estimated values of 18.1 kg, 21.3 kg and 2.2 kg for ALST, FM, and BMC, respectively. Their true measured values (DXA) were 18.2 kg, 20.8 kg, and 2.2 kg. If the equation is applied to an older adult woman “B” with values of weight (58.6 kg), HAS (81.5 cm), TriSK (26 mm), and sex (1) the estimated values for ALST, FM, and BMC would be: 13.1 kg, 23.5 kg and 1.9 kg, correspondingly. As noted, the values are close to the measured DXA values for ALST (13.2 kg), FM (23.4 kg), and BMC (2.0 kg). These values can be compared with the reference values National Health and Nutrition Examination Survey (NHANES) [60] and be useful for many applications in clinical and field practice. For example, using the criteria proposed by the FNIH (ALST cutoffs < 19.75 for men and < 15.02 for women) we can classify both older adults with low ALST and probable sarcopenia [61]. These findings are highly relevant as they allow permanent following/monitoring of excessive accumulation of FM, and declines in BMC and ALST, as risks to older adults throughout the life course [62, 63]. Thus, keeping the balance rate of fat, muscle and bone is essential to preserving metabolic homeostasis, and health status and positively contributes to successful aging [56]. For this reason, the assessment of BC in older adults is critical and could be an additional preventive strategy for age-related diseases [56], which may result in sarcopenia [4, 6, 64], osteoporosis [65] sarcopenic obesity [43] osteosarcopenic obesity (2) and osteosarcopenia [66]. This should impair muscle strength, and functional capacity, as well as greater morbidity and mortality in older adults [67]. Therefore, the current prediction equations could increase the available options for the estimation of BC in older adults. To ensure dissemination and accessibility, an assessment of the main body components based on our predictive models can be found in an excel file (Additional file 1) at the following link (http://posgraduacao.eerp.usp.br/files/Model_BodyComposition_OlderAdults.xlsx). Lastly, future studies should evaluate the efficiency of these equations applied in longitudinal and intervention studies.

## Conclusion

Our findings demonstrated that the anthropometric prediction equations developed in this study provide a reliable, practical, and low-cost instrument to assess the components that most change during the aging process. These results suggest that the equations can be valid alternatives and reliable information about BC in older adults since the internal validation method PRESS presented high internal validity, high coefficients of determination, and low prediction errors.

## Data Availability

The datasets generated during and/or analyzed during the current study are available from the corresponding author upon reasonable request.

## Abbreviations

BC: Body composition
SMM: Skeletal muscle mass
FM: Fat mass
FFM: Fat-free mass
BMC: Bone mineral content
LST: Lean soft tissue
ALST: Appendicular lean soft tissue
PRESS: Predicted residual error sum of squares
HAS: Half-arm span
TriSK: Triceps skinfold
SEE: Standard error to estimate
BD: Body density
DXA: Dual-energy X-ray absorptiometry
MRI: Magnetic resonance imaging
Q^2^_PRESS_: Press coefficient of determination
S_PRESS_: Press standard error of estimate
CI: Confidence interval
SD: Standard deviation
UL: Upper limit
IL: Inferior limit
Min: Minimum
Max: Maximum
